# Adult Brainstem Glioblastoma Multiforme: Long-term Survivor

**DOI:** 10.7759/cureus.434

**Published:** 2015-12-25

**Authors:** Zachary R Barnard, Doniel Drazin, Serguei I Bannykh, Jeremy D Rudnick, Ray M Chu

**Affiliations:** 1 Neurosurgery, Cedars-Sinai Medical Center; 2 Pathology, Cedars-Sinai Medical Center; 3 Neurology, Cedars-Sinai Medical Center

**Keywords:** glioblastoma, neuro-oncology, brainstem glioma

## Abstract

Adult, malignant brainstem gliomas are rare entities that often cause treatment conundrums due to the difficulty of surgical resection and, therefore, the absence of pathological diagnosis. This leads to a reliance on radiological imaging for diagnosis, which can often be unreliable. These shortcomings have made the treatment of brainstem gliomas challenging with unpredictable outcomes. The mainstay of treatment consists of chemotherapy and radiation; however, recurrence is inevitable. Predicting outcomes has been the major difficulty in treating these patients as adult malignant brainstem gliomas Grade II have a median survival between five to seven years while Grades III and IV are between 10-17 months (with some studies showing significantly longer survival in Grade III). Here, we present the case of a patient with the pathologic diagnosis of a right brachium pontis glioblastoma who had a remarkable survival of 73 months, whereas the expected median survival for these patients is 10-17 months.

## Introduction

Adult brainstem gliomas are a very different disease entity when compared to pediatric brainstem gliomas and present significant management challenges due to their rarity and absence of clear diagnostic classifications. While gliomas represent 40-60% of all primary brain tumors, brainstem gliomas only represent about 1-2% with malignant high-grade brainstem gliomas (WHO Grade III and IV) representing only a minority [1-5]. Even with a pathological diagnosis, it has been difficult to distinguish WHO Grade III from IV. This has led to only a limited number of case series describing experiences with treatment and associated prognosis. Given their location, biopsy or surgical resection has been controversial due to the relatively high risk to delicate structures. For this reason, treatment decisions have relied mostly on radiologic appearance of serial MRIs.

Four typical delineations are used for classification of brainstem gliomas based on MRI: diffuse intrinsic low-grade gliomas, enhancing malignant gliomas, focal tectal gliomas, and exophytic gliomas [6]. The enhancing malignant gliomas have classically included both WHO Grades III and IV gliomas. This presents a problem as median survival has been observed in some studies to be different between the WHO Grade III anaplastic astrocytomas of the brainstem (77.0 months) and the WHO Grade IV glioblastomas (12.1 months) [1]. The caveat to many of these series is that they have very limited number of patients due to the rarity of the condition.

Here, we discuss a case of a man with a clinical, radiographic, and histological diagnosis of a brainstem glioblastoma who underwent surgical resection, chemotherapy, and radiation with an overall survival of 73 months. This case aims to illustrate the difficulties of predicting brainstem glioma outcomes and alludes to the necessity for better biomarkers.

## Case presentation

**Figure 1:**
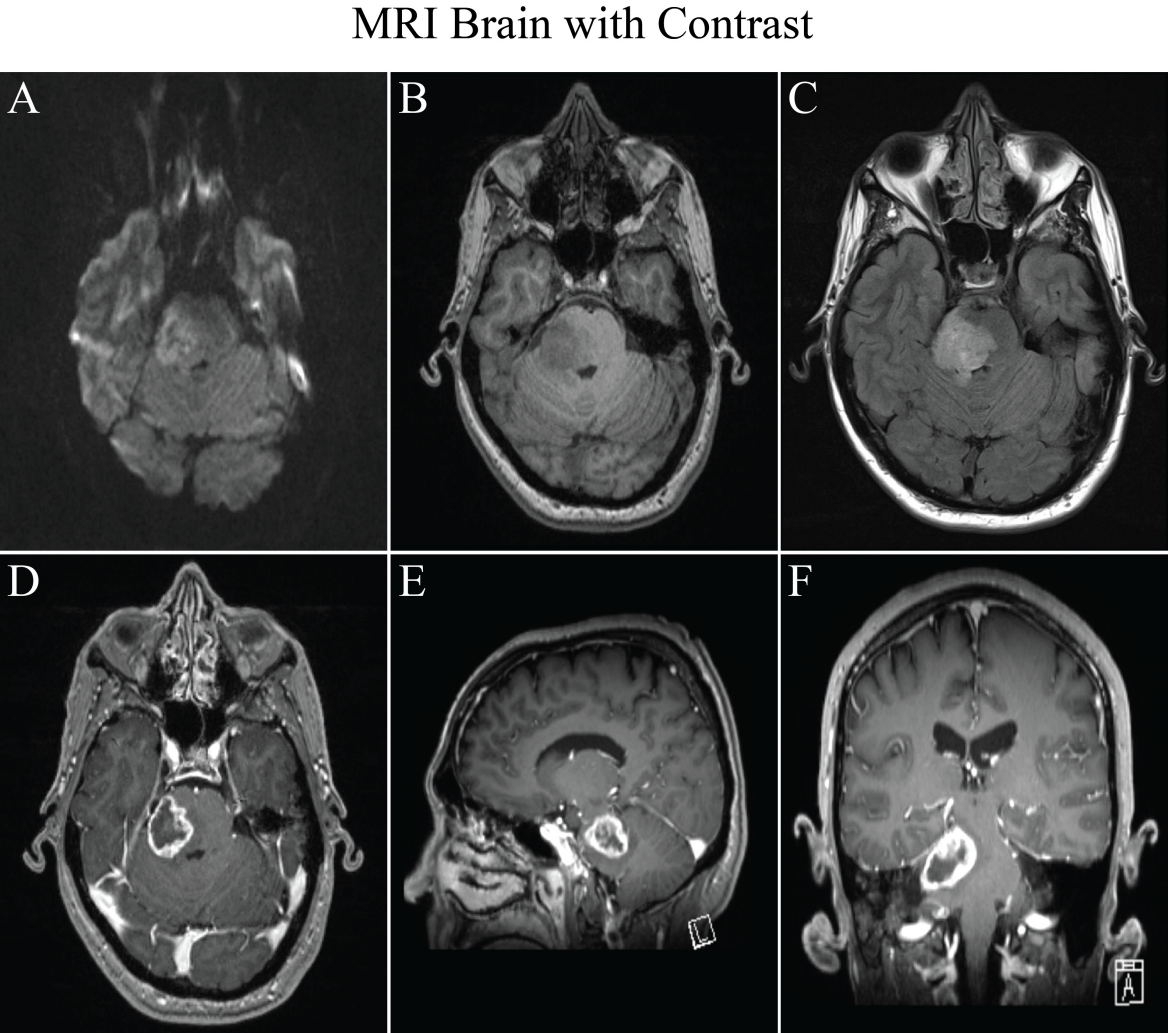
MRI brain with contrast at diagnosis A. Diffusion weighted imaging displaying no restricted diffusion. B. T1 pre-contrast. C. T2 FLAIR. D. Axial T1 post-contrast. E. Sagittal post-contrast. F. Coronal post-contrast.

We present a case of a 55-year-old, right-handed male who initially presented to his primary care physician 6 years prior with a 2-week history of double vision, progressive headaches, neck stiffness, toothaches, and subsequently right facial numbness. Given his unremarkable dental workup, he underwent an MRI of the brain that showed a right 2.9 cm (anterior-posterior) by 2.5 cm (transverse) by 2.0 cm (superior-inferior) brachium pontis heterogeneously ring-enhancing mass with associated edema (Figure 1). He was referred to a neurosurgeon, and surgical resection was offered.

**Figure 2:**
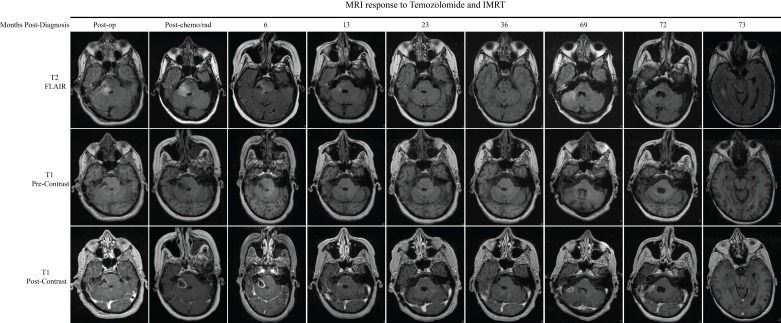
MRI response of tumor growth to temozolomide and radiation therapy Serial MRI scans of the brain showing response of the lesion to chemotherapy and radiation. Recurrence in the right cerebellum is noted at 69 months. Minimal ependymal enhancement can be seen in the right temporal horn of the ventricle at 73 months.

The patient proceeded with a right retrosigmoid craniotomy for resection of his tumor. Intra-operatively, the lesion was noticed to be cystic with hypervascularity. To prevent a postoperative neurological deficit, a subtotal resection was performed, although postoperative imaging revealed a significant resection (Figure 2). Postoperatively, he did well and was discharged home with a dexamethasone taper.

**Figure 3:**
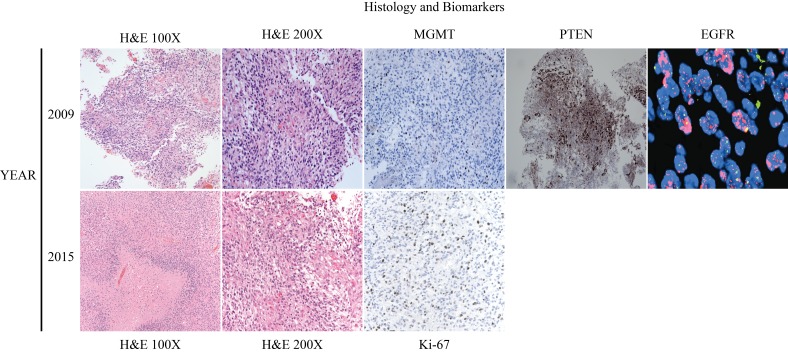
Histology and Biomarkers. This figure displays the histology, immunohistochemistry, and FISH done on the initial resection tissue in 2009 and the recurrent tissue in 2015. 2015 MGMT, PTEN, and EGFR were the same as 2009 and thus not included.

Pathological specimens were microscopically reviewed by way of histology, immunohistochemistry (MGMT, PTEN, Ki-67), and fluorescence in-situ hybridization (EGFR). Histologically, the tumor displayed hypercellularity, microvascular proliferation, and areas of pseudopalisading necrosis, leading to the diagnosis of glioblastoma multiforme, WHO Grade IV (Figure 3). Other supportive and prognostic biomarkers exhibited amplification of EGFR, PTEN expression, and low MGMT expression (Figure 3). At this time, IDH1 and IDH2 were not routinely used for prognosis.

After a multispecialty discussion, it was determined that he would start adjuvant chemotherapy and radiation. Two weeks postoperatively, he started a six-week course of concurrent temozolomide and radiation. Temozolomide was started at a daily dose of 75 mg/m^2^ for a six-week duration, along with radiation, totaling of 60 Gy. Over this timeframe, he continued on low-dose dexamethasone to reduce the edema associated with radiation. After completing his initial chemotherapy and radiation course, a repeat MRI of the brain was done that showed no change in the size of the enhancing portion of his residual tumor with some associated edema (Figure 2).  

Over the subsequent two years, he continued monthly cycles of temozolomide of 200 mg/m^2^ daily for five days for a total of 23 cycles and had MRIs every two to three months to track progression. By 36 months, the residual enhancing portion in the right brachium pontis had regressed significantly (Figure 2).

Regrettably, 69 months after his initial diagnosis, new enhancement was seen along the resection tract in the right cerebellum (Figure 2). After a long discussion, he decided to have a repeat surgical resection. Pathological specimens showed recurrent glioblastoma with similar histological and biomarkers to his initial resection as well as IDH1 negative and Ki-67 of 30% (Figure 3). Nine days postoperatively, he represented with headaches, severe incisional pain, and fevers. Lumbar puncture showed significant leukocytosis concerning for meningitis. He went for wound incision and drainage and was started on broad-spectrum antibiotics. His intraoperative and CSF cultures were consistent with methicillin-sensitive *Staphylococcus aureus *and he was changed to oxacillin for a total of four weeks followed by two weeks of oral amoxicillin.

After completing his antibiotics therapy, he underwent a temozolomide re-challenge with 2 cycles of 125 mg/m^2^ daily, and a stereotactic radiosurgery re-irradiation to the resection cavity over five days.

Two months later, he was admitted to the hospital for confusion and a lumbar puncture showed mildly elevated WBC concerning for recurrent meningitis. He was placed on ceftriaxone IV and no cultures grew bacteria; however, antibiotics were started 48 hours prior to lumbar puncture. Repeat MRI of the brain showed new strokes and slight ependymal enhancement in the right temporal horn which may have signified leptomeningeal carcinomatosis (Figure 2). Given his poor mental status and deterioration of activities of daily living, his family pursued hospice care and he passed away 73 months after his initial diagnosis.

## Discussion

In this case report, we describe a middle-aged man diagnosed with a brainstem glioblastoma who, with standard therapy of surgical resection, chemotherapy, and radiation, survived for 73 months. This is one of the longest survivals documented in the literature. This case is a perfect illustration of the limitations associated with current diagnosis and treatment of adult brainstem gliomas. Based on radiographic appearance and histological grade, the patient should have had a median survival of 10-17 months, and yet, he survived for 73 months, similar to a low-grade brainstem glioma [1]. It is prudent that we question whether or not our classic understanding and treatment of supratentorial low and high-grade gliomas are reliably applicable to low and high-grade brainstem gliomas. If they are not applicable, what makes the supratentorial low and high-grade gliomas different from the low and high-grade brainstem gliomas? Is it the tumor micro-environment or is it characteristics of the tumor itself?

The current radiology classification may be helpful to guide treatment, but it should not be thought of as a replacement for histological grading and molecular characterization. As more targeted therapies are developed, the need for tissue diagnostics becomes increasingly important. Correlation between MRI imaging characteristics and histological grade has been estimated as low as 67% [7]. We should advocate for surgical biopsy or resection when deemed safe, as this will allow for tissue diagnosis and directed therapy.

The patient’s overall survival could be explained by the fact that he had aggressive surgical resection, whereas many of these tumors have only a biopsy followed by adjuvant therapy. Also, given his low expression of MGMT, it would be expected that he would have a favorable response to temozolomide.

## Conclusions

In this case, we exemplify the essential need for tissue diagnosis in brainstem gliomas without which adequate treatment will be difficult. However, despite adequate tissue diagnosis, prognostication may still be challenging due to the ambiguous outcomes related to tumor grade and other current diagnostic tools. Ultimately, future biomarker identification that correlates with treatment effect and survival is necessary to improve diagnosis and prognosis associated with brainstem gliomas.
